# Neuromyelitis optica spectrum disease coexisting with subacute combined degeneration: a case report

**DOI:** 10.1186/s12883-022-02870-4

**Published:** 2022-10-04

**Authors:** Yixuan Zeng, Runtao Bai, Yanxia Zhou, Lijie Ren

**Affiliations:** Department of Neurology, The First Affiliated Hospital of Shenzhen University, Shenzhen Second People’s Hospital, Shenzhen, China

**Keywords:** Subacute combined degeneration, Neuromyelitis optica spectrum disorders, Vitamin B12 deficiency, Aquaporin 4, Magnetic resonance imaging

## Abstract

**Background:**

Subacute combined degeneration (SCD) is a demyelinating disease characterized by vitamin B12 deficiency related segmental degeneration of the dorsal or lateral columns of the spinal cord. However, few cases have been reported as a comorbidity of SCD and neuromyelitis optica spectrum disease (NMOSD).

**Case presentation:**

Herein, we describe a female patient (61-year-old) who had sensory deficits, paresthesia, and weakness of the distal extremities for over 2 months. She then received an initial diagnosis of SCD with typical inverted “V-sigh” hyperintensities over the posterior aspect of the spinal cord in magnetic resonance imaging (MRI - T2-weighted imaging), as well as megaloblastic anaemia in blood examinations. From the past history, there was no evidence of a dietary deficiency or gastric abnormalities. However, traditional treatment with vitamin B12 supplementation was ineffective. Hence, a demyelinating antibody examination showed that she had antibodies targeting aquaporin 4 (AQP4) in both the cerebrospinal fluid and serum, leading to the diagnosis of NMOSD. Her clinical symptoms were obviously improved after treatment with intravenous glucocorticoids.

**Conclusion:**

People who have nutritional deficiency or altered gastrointestinal function are more likely to develop SCD. This case raises the awareness that the poor therapeutic effects of simple vitamin B12 supplementation could be explained by immunoreactions against AQP4. A better recognition will be of great importance for the correct diagnosis of the comorbidity, as well as for essential treatment and even a better prognosis.

## Background

Subacute combined degeneration, short as SCD, is a vitamin B12 deficiency related treatable neurological demyelinating complicatio. Although the mechanism of demyelination that is caused by cobalamin deficiency is not entirely clear, SCD often occurs in elderly individuals or people with malabsorptive disorders and inadequate vitamin B12 intake or bioavailability. Neuromyelitis optica spectrum disease (NMOSD) is an antibody-mediated demyelinating disease of the central nervous system. NMOSD often presents as myelitis (long segments of spinal cord inflammation). However so far there have been few reports of SCD coexisting with NMOSD. Moreover, the specific treatment regimen and duration of treatment can vary depending on the coexistence of the two diseases. Here we reported an older woman diagnosed with SCD coexisting with NMOSD and described the clinical manifestations, magnetic resonance imaging (MRI) presentations, treatment, and outcomes. Furthermore, we highlighted the importance of immune therapy with glucocorticoids in this patient, as well as the importance of antibody testing in SCD patients.

## Case presentation

A 61-year-old woman presented with numbness and weakness in all of her extremities for over 2 months. She then went to the community clinic and received treatment of oral vitamin B12 (1 mg per day) with the guidance of community doctors. She felt slight improvement of the numbness when first got oral vitamin B12 treatment for about a week. However, after the orally treatment of vitamin B12 for one month, her symptoms worsened over time and interfered with her daily life. And then she was admitted to department of neurology in our hospital. The patient had Hashimoto’s thyroiditis and hypertension, as well as good compliance with the hypotensor. In addition, she had a good dietary intake with a negative history of alcohol, cigarettes, illicit drugs, or gastrointestinal surgery. She was conscious and fluent in speech when she arrived at our clinic. A neurological examination showed no abnormalities in cranial nerves. A marked increase in the deep tendon reflexes, mild weakness in the limbs (grade 4), and impaired vibration sensation and joint position were detected on the neurological examination. Neither Babinski’s sign nor Romberg’s sign were detected at that time. The scores of the Mini-Mental State Examination (MMSE) and Montreal Cognitive Assessment (MoCA) were both 21 out of 30. Moreover, macrocytic anaemia was demonstrated by the laboratory tests. There was a decrease in red blood cells (RBCs) (2.47 × 10^12/L, reference range: 4.30–5.80 × 10^12/L) and haemoglobin (Hb) (106 g/L, reference range: 130–175 g/L), which were accompanied by an increase in MHC (42 pg, reference range: 27–34 pg), MCHC (360 g/L, reference range: 320–360 g/L), and MCV (116.7 fL, reference range: 82–100 fL). Furthermore, a lower vitamin B12 (147 pmol/L, reference range: 211–911 pmol/L) level was detected even after supplementary treatment with vitamin B12. The level of serum homocysteine (Hcy) (31.6 μmol/L, reference range: 4–15.4 μmol/L), an indicator of the function of vitamin B12 deficiency at the cellular level, was out of the normal range.

**Fig. 1:**
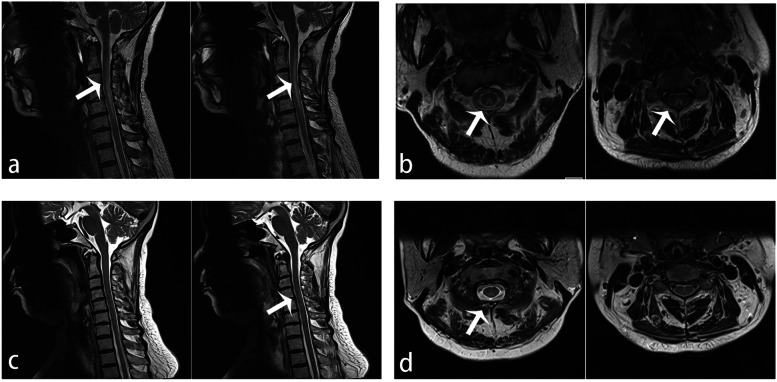
Spinal cord MRI. **a** Sagittal T2-weighted imaging showed long segmental hyperintensities from C2 to C6 of the spinal cord. **b** Inverted “V-sign” hyperintensity on axial T2-weighted imaging within the dorsal cervical spinal cord. **c** After 3 months of continuous immune therapy, there was a decreased signal on axial T2-weighted imaging, both horizontally and longitudinally. **d** After 3 months of continuous immune therapy, the inverted “V-sign” hyperintensity faded on axial T2-weighted imaging

The cerebrospinal fluid (CSF) showed normal leukocyte, chloride, glucose, and protein results. Anti-intrinsic factor antibody and tumour markers (CEA, AFP, CA125, CA724, CA19–9, and CA15–3) were all unremarkable. Nerve conduction studies and electromyogram (EMG) were normal. In spinal cord T2-weighted MRI, long segmental hyperintensities involving the posterior columns of the spinal cord were observed in sagittal images (C2 to C6) (Fig. 1a), whereas a typical inverted “V-sign” was observed in axial images (Fig. 1b). No abnormality was found in the brain MRI scan. She was initially diagnosed with SCD for unknown reasons. As she was benefits from oral vitamin B12 supplementation at the very beginning, a high dose of supplementary intramuscular vitamin B12 injections (1.5 mg per day) was given immediately after she came to our department. And at the same time, a neuromyelitis optica (NMO) antibody test was then performed, as there was a longitudinally extended lesion on the sagittal MRI as well as the poor curative effect of vitamin B12 supplementation alone. The results showed that antibody targeting aquaporin 4 (AQP4) was positive in both the serum (12.86 u/ml) and in the CSF (7.98 u/ml), and a myelin-oligodendrocyte glycoprotein (MOG) antibody test was negative. She was then diagnosed with NMOSD coexisting with SCD.

Subsequently, she received intravenous methylprednisolone treatment (500 mg/day for 3 days, 250 mg/day for 3 days, 120 mg/day for 3 days). Symptoms of weakness were improved via the intravenous methylprednisolone treatment. After intravenous methylprednisolone, she continued the oral prednisolone treatment for 6 months. The course of intramuscular vitamin B12 injections for 1 month with a following daily oral vitamin B12 supplementation treatment (1 mg per day) for 6 months. 3 months after discharged, except for slight numbness in the fingers, symptoms of paraesthesia and limb weakness had resolved. Additionally, her anaemia improved (RBC 3.48 × 10^12/L and Hb 127.0 g/L), and the vitamin B12 level in serum increased to more than the maximum measurable value. Signal abnormalities observed on the MRI also improved, both horizontally and longitudinally (Fig. 1c, d). During her next 6-month follow-up, her symptoms were completely relieved. In addition, no tumour signs were discovered during the follow-up period.

## Discussion and conclusions

SCD is an uncommon type of myelopathy. NMOSD is also an uncommon antibody-mediated disease of the central nervous system (CNS). Here we reported a rare clinical case of simultaneous NMOSD and SCD with a subacute onset of numbness and weakness in all of her extremities [1]. A typical imaging finding of SCD is an increased signal intensity (inverted “V” sign) in the dorsal columns of the cervical cord in MRI T2-weighted imaging [2]. One of the specific presentations of NMOSD is longitudinally extensive transverse (but not selective) myelitis [3]. The relationship between SCD and NMOSD remains unclear.

A retrospective analysis of aquaporin-4 antibody-positive NMSOD patients by the Neurosciences Group at Oxford University found that 46.2% of the confirmed patients had low vitamin B12 levels (mean: 150 pmol/L; standard: 300 pmol/L) without positive anti-intrinsic factor antibody [4]. This finding can be explained by the fact that the cell membranes of gastric parietal cells are also rich in AQP4 expression; thus, gastric parietal cells may also be damaged due to the immune response. As a result, the low level of vitamin B12 in NMOSD patients may be caused by damage to the humorall immune response to gastric parietal cells. One hypothesis is that SCD caused by vitamin B12 deficiency may be one of the clinical manifestations of NMOSD. The systemic immune response associated with AQP4 leads to a diversity of clinical symptoms. Therefore, NMOSD always coexists with other humorall autoimmune diseases, such as connective tissue disease, myasthenia gravis, or celiac disease [5, 6].

Moreover, as an intermediate of cobalamin metabolism, elevation of Hcy can be used to confirm B12 deficiency [7]. Hcy should be measured in patients with suspected clinical B12 deficiency, especially when serum B12 levels are either normal, borderline, or accompanied by macrocytic anaemia [8]. High Hcy levels can increase the production of reactive oxygen species (ROS) to induce oxidative stress and stimulate the N-methyl-D-aspartate (NMDA) receptor to promote excitotoxicity [9]. Hence, Hcy will lead to neuronal damage, as the nervous system is particularly sensitive. Serum Hcy levels can also be used as an independent predictor of recurrence and poor prognosis for NMOSD patients [10]. For most patients with SCD or NMOSD, MRI results are always delayed behind the clinical presentation; therefore, long-term follow-up is needed.

In conclusion, even if it is easy to diagnose SCD by detecting vitamin B12 levels and by utilizing spinal cord MRI, it is usually difficult to determine the real pathogeny of vitamin B12 deficiency. Nutritional supplementation alone may lead to the delay of effective treatment and the aggravation of SCD. Therefore, AQP4 antibody examination is suggested for SCD patients with no obvious cause and poor effects of routine treatment. Moreover, immunotherapy is necessary in AQP4-positive cases [11, 12]. Finally, a long-term prognosis is as important as the determination of the underlying aetiology for SCD patients.

## Data Availability

Data has not been made accessible in the interest of protecting patient’s privacy.

## Abbreviations

AQP4: Aquaporin 4
CNS: Central nervous system
CSF: Cerebrospinal fluid
Hb: Hemoglobin
Hcy: Homocysteine
MMSE: Mini-Mental State Examination (MMSE)
MoCA: Montreal Cognitive Assessment (MoCA)
NMDA: N-methyl-D-aspartate
NMO: Neuromyelitis optica
NMOSD: Neuromyelitis optica spectrum disorders
MOG: Nyelin-oligodendrocyte glycoprotein
MRI: Magnetic resonance imaging
RBC: Red blood cell
ROS: Reactive oxygen species
SCD: Subacute combined degeneration
